# Late stroke after transcatheter aortic valve replacement: a nationwide study

**DOI:** 10.1038/s41598-021-89217-0

**Published:** 2021-05-05

**Authors:** Henrik Bjursten, Bo Norrving, Sigurdur Ragnarsson

**Affiliations:** 1 Department of Cardiothoracic Surgery, Skåne University Hospital, Lund University, 221 85 Lund, Sweden; 2 Department of Clinical Sciences Lund, Neurology, Skåne University Hospital, Lund University, Lund, Sweden

**Keywords:** Cardiology, Cardiac device therapy, Interventional cardiology

## Abstract

Transcatheter aortic valve replacement (TAVR) is a rapidly growing field. Short-term safety and efficacy of these procedures have been studied extensively. However, little is known about the safety of these devices over time. Stroke is one feared long-term complication, and an increased stroke rate could affect guidelines for treating both the aortic stenosis and choosing antithrombotic therapy after TAVR. The primary objective was to study the incidence of stroke up to 8 years after TAVR implantation, comparing it with the risk of stroke in the general population. Secondary objectives were to study risk factors for late stroke and to study outcomes after stroke. A nationwide, all-comers study of patients who underwent TAVR in Sweden 2008–2018 was performed. The study was based on data from three national registries: a TAVR registry, a stroke registry, and a diagnosis registry. The main outcome was stroke incidence 30-days or more after TAVR implantation and was compared to a standardized incidence. The annual risk for stroke varied between 2.0% and 3.1% as compared to 1.5% and 1.9% in an age- and sex-matched cohort. Risk factors for developing stroke were reduced renal function, diabetes, history of stroke, age, and male sex. The 1-year mortality after stroke was 44%. This study demonstrated an increased rate of stroke after TAVR, but the findings suggest that this can in part be attributed to the group’s higher frequency of pre-disposing risk factors.

## Introduction

Transcatheter aortic valve replacement (TAVR) is a rapidly growing therapy, and the expansion of the procedure has been justified by several well-documented randomized trials^1–5^. These studies have demonstrated good short-term and medium-term outcomes compared to surgical aortic valve replacement (SAVR). With the expansion of TAVR, an increased number of patients are treated outside the inclusion criteria, and patients are living longer than study follow-up periods^6^. Therefore, the safety of TAVR valves over time in a real-world setting is of great interest. Particularly relevant are life-threatening late complications such as prosthetic valve endocarditis, stroke, and valve dysfunction, as an increase in late incidence could affect treatment choice in patients with aortic stenosis. TAVR valves have a geometry that differs from surgical valves with more stent material that extends to the left ventricular outflow tract (LVOT) and ascending aorta. In addition, after a TAVR implantation the native valve is immobilized around the stent-frame, and both these structures could act as sources of emboli^7^. Therefore, an increase in late complications is a possibility and must be studied.

Stroke is a well-documented complication in the early period following TAVR, with 30-day stroke rates spanning between 1.0% and 5.5% in newer studies^1,2,4,8–11^, and 1-year stroke rates spanning between 4.3% and 8.2%^1,2,4,11,12^, where some of these numbers are based on randomized studies and some on real-world data. Looking beyond the 1-year and especially 2-year mark, little is known about the risk of developing a stroke after receiving TAVR and whether the risk is higher than it is in the general population.

The aim of the present nationwide, all-comers study was, therefore, to study the risk of developing stroke up to 8 years after a TAVR procedure as well as to study stroke subtypes, survival after stroke, and predictors for stroke.

## Methods

### Study design

This is a retrospective, nationwide follow-up study of all patients who underwent TAVR in Sweden from January 2008 to September 2018. The dataset and data sources have been described earlier^13^. The initial data source was the national TAVR registry SWENTRY (SWEdish traNscatheter cardiac intervention regisTRY), which is a sub-registry of SWEDEHEART (Swedish Web-system for Enhancement and Development of Evidence-based care in Heart Disease Evaluated According to Recommended Therapies) and contains information on all TAVR procedures performed in the country^14^. Of the 4336 implantations in the registry, 28 were a TAVR-in-TAVR, and we used the first implantation in each case as the index procedure for this analysis. An additional 69 patients had a stroke during the procedure, and 34 patients had a stroke during the first 30 days, leaving 4205 patients for the final analysis.

In all registries, patients are identified by a unique personal identification number, which is used for all governmental and healthcare interactions. This made it possible to cross-reference the SWENTRY registry with other registries and also allowed us to verify date of death for deceased patients. The study was approved by the Regional Ethical Review Board in Lund, Sweden (registration number 2017/995). The study was performed according to the declaration of Helsinki and STROBE guidelines and compliant with local and national slaws and regulations. The study was registered in clinicaltrials.gov under the identifier NCT04086836.

### Definition of stroke

The Riksstroke registry and the National Patient Registry (“Patientregistret”, NPR) were used to establish the diagnosis of stroke^15,16^. The Riksstroke registry is a nationwide registry with a very high coverage rate of all strokes (see Supplementary Information). The NPR contains information on all admissions to hospitals in Sweden, and data entry is mandatory by law. A main discharge diagnosis according to the International Statistical Classification of Diseases and Related Health Problems (ICD-10) is required and the registry allows for multiple secondary diagnoses. To identify patients with stroke we selected the ICD-10 codes I61 (intracerebral haemorrhage) and I63 (cerebral infarction) as their primary diagnosis.

### Background risk

For calculating a standardized age- and sex-specific incidence of stroke in the general population, we used national population records together with data from Riksstroke for 2017, making it possible to estimate standardized risk for stroke based on age and sex^17^. The numbers were then used to assign an age- and sex-specific standard incidence of stroke to each patient for every year the patient was in the study (with a parallel increase in year for risk category). This procedure could not take into account any co-morbidities and thus created a control group that most probably was healthier than the TAVR-group. A mean background stroke risk was calculated for every year post-TAVR up to 8 years (see Supplementary Information).

### Variables in the model

Variables in the SWENTRY registry (Supplementary Table 1) were based on the EuroScore and VARC-2 definitions^18,19^. The estimated glomerular filtration rate (eGFR) was calculated from creatinine according to the Chronic Kidney Disease Epidemiology Collaboration’s formula^20^. The different valve models were divided into two groups (Supplementary Table 2): self-expandable valve (SEV) or balloon expandable valve/mechanically expandable valve (BEV/MEV).

### Patient and public involvement

As this was a retrospective study, no direct Patient or Public involvement was done. However, as many patients ask about the risks associated with a TAVR procedure, this concern is addressed in this study.

### Statistical analysis

Kaplan–Meier curves were used to illustrate accumulated stroke incidence and survival after stroke. A Cox proportional hazard model was used to find predictive factors associated with stroke during follow-up from 30 days and more. Data was complete in all but five cases (none of which were diagnosed with stroke), and these cases were excluded in the multivariable analysis but were included in the description of the population. Variables were selected if significant (p < 0.1) in univariable Cox analysis or had clinical interest (history of atrial fibrillation, new atrial fibrillation after the procedure, porcelain aorta, and valve type). A Backwards Stepwise exclusion was used with a p < 0.1 to stay in the model. Cox regression modelling was performed starting at 30 days after TAVR. After the final model was built, the excluded risk factors were added one by one to see if the model changed significantly. Martingale residuals were used to assess goodness of fit, and a Harrell’s C-index was used to estimate model strength. The proportional hazard assumption in the Cox model was tested by Schoenfeld residuals. Only the first episode of stroke was analysed. Student’s t-test, Chi^2^-test, or Mann–Whitney U-test was performed depending on the distribution of data. Data is presented as mean ± standard deviation (SD), number (%), or median with interquartile range (IQR). A p-value < 0.05 was considered statistically significant. Data analyses were performed using the SPSS package version 25 (IBM Corp. Armonk, NY, USA) and Stata version 14 (StataCorp LLC, College station, TX).

## Results

Patients were followed for a median 24.5 (IQR 11.4–42.7) months, yielding a total of 10,467 patient-years for the study. We identified 228 patients who had suffered a stroke 30 days or more after TAVR in Riksstroke, and an additional 7 patients were found in the NPR with a main diagnosis of stroke, totalling 235 stroke patients for the analysis. The cohort is described in Supplementary Table 1.

**Figure 1:**
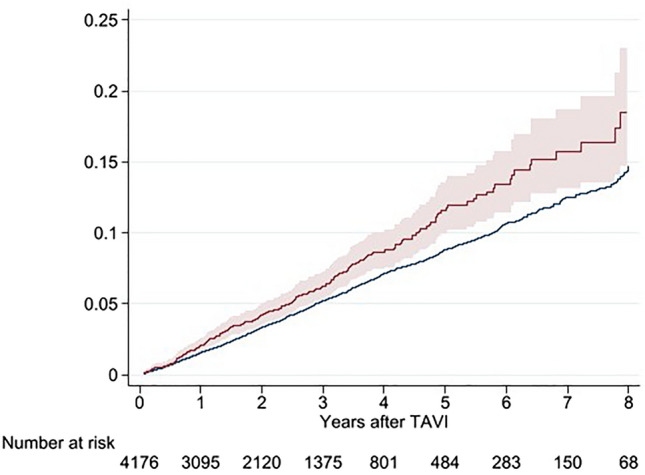
Accumulated risk for stroke up to 8 years after TAVR presented as a Kaplan–Meier failure function with 95% CI. Blue indicates accumulated standardized incidence calculated for this cohort of patients. Curve truncated at 8 years due to small numbers.

**Figure 2:**
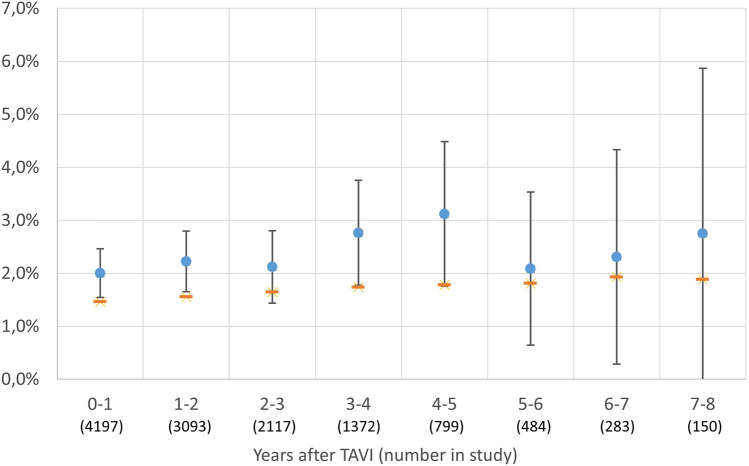
Risk for stroke per year post-TAVR with 95% CI. Red bar indicates standardized incidence calculated for this cohort of patients.

The mean annual incidence of stroke during the follow-up varied between 2.00% (95% CI 1.54–2.46%) and 3.12% (95% CI 1.75–4.48%) compared to the standardized incidence that ranged between 1.46 and 1.93% (Figs. 1, 2, Supplementary Table 3).

In univariable analysis, eGFR < 30 ml/min/1.73 m^2^, age, male sex, history of stroke, diabetes, mildly reduced LVEF, peripheral vascular disease, and dialysis after procedure were predictors for late stroke, whereas valve-in-valve (TAVR in SAVR) procedure was associated with a lower risk for stroke (Table 1 and Supplementary Fig. 1). In multivariable analysis, eGFR < 30 ml/min/1.73 m^2^ (HR 2.07 [96% CI 1.39–3.11]), diabetes (1.59 [1.19–2.12]), history of stroke (1.48 [1.07–2.05]), age (HR per year 1.03 [1.00–1.05]), male sex (1.28 [0.99–1.67]) were predictors of late stroke, whereas valve-in-valve procedure (0.09 [0.01–0.62]) was associated with less incidence of stroke (Table 1). This model yielded a Harrell's C-index of 0.64.

**Table 1:** Risk factors for developing a stroke after a successful TAVR.

	Univariable		Multivariable	
	p-value	HR (95% CI)	p-value	HR (95% CI)
eGFR < 30 ml/min/1.73 m^2^	0.001	2.03 (1.36–3.03)	< 0.001	2.07 (1.39–3.11)
Diabetes	0.001	1.59 (1.20–2.09)	0.002	1.59 (1.19–2.12)
History of stroke	0.004	1.60 (1.16–2.21)	0.018	1.48 (1.07–2.05)
Age (per year)	0.066	1.02 (1.00–1.04)	0.010	1.03 (1.00–1.05)
Male	0.096	1.24 (0.96–1.61)	0.058	1.28 (0.99–1.67)
Valve-in-valve	0.017	0.09 (0.01–0.65)	0.015	0.09 (0.01–0.62)
Post-procedural atrial fibrillation	0.111	1.78 (0.88–3.60)		
Moderately depressed LV function	0.079	1.34 (0.97–1.86)		
Porcelain aorta	0.299	1.36 (0.76–2.45)		
Peripheral vascular disease	0.056	1.34 (0.99–1.82)		
New dialysis	0.098	3.24 (0.81–13.1)		
SEV	0.955	1.01 (0.78–1.30)		
Atrial fibrillation before procedure	0.803	0.97 (0.74–1.27)		

Univariable analysis include variables with a p < 0.100 and variables that could be of clinical interest. The multivariable analysis was constructed from a backwards elimination method.

*eGFR* estimated Glomerular Filtration rate, *SEV* self-expandable valve.

There was no difference between BEV/MEV and SEV in univariate analysis (HR 1.01 [0.78–1.30], p = 0.955). This variable was forced into the multivariable analysis and still reached significance.

Of the 235 patients with stroke, 210 strokes were classified as ischemic stroke (IS), 24 were classified as hemorrhagic stroke (HS), and one stroke was unable to be classified as ischemic or hemorrhagic. The stroke occurred on a median 20.6 (IQR 9.9–38.3) months after the TAVR procedure, with no significant difference between IS and HS (Table 2). Almost half of the patients (47.7%) had atrial fibrillation when they were admitted for their stroke, and no significant difference was seen between IS and ICH in rate of atrial fibrillation (46.7% vs 54.2%, p = 0.486). Consciousness at arrival was documented. While the majority (82.1%) were fully conscious, 5.1% were unconscious at arrival with an overrepresentation of HS compared to IS (25.0% vs 2.9%, p < 0.001), and 8.1% had a reduced consciousness, which also was seen more often in HS than IS (16.7 vs 7.1, p < 0.001). An additional 4.7% were not classified (Table 2).

**Table 2:** Characteristic of patients suffering a stroke.

	All	IS	HS	p
Time from TAVR (months)	20.6 (9.9–38.3)	19.7 (9.7–37.3)	30.0 (10.0–44.4)	0.253
Age	83.8 (7.5)	84.1 (7.5)	81.5 (7.5)	
Male sex	125 (54.8%)	107 (52.7%)	17 (70.8%)	0.092
Hospitalization total (days)	14.2 (13.2)	14.4 (13.5)	13.0 (10.3)	0.886
TIA/Amaurosis Fugax	27 (11.9%)	24 (11.9%)	3 (13.0%)	0.871
Previous stroke	54 (23.8%)	48 (23.8%)	6 (25.0%)	0.893
Atrial fibrillation	112 (47.7%)	98 (46.7%)	13 (54.2%)	0.486
Hypertension	174 (77.3%)	159 (79.1%)	14 (60.9%)	0.048
Diabetes	81 (35.8%)	69 (34.2%)	11 (47.8%)	0.194
On lipids	114 (50.4%)	103 (51.2%)	10 (41.7%)	0.375
On ASA	107 (47.3%)	100 (49.8%)	7 (29.2%)	0.056
On clopidrogel	35 (15.5%)	32 (15.9%)	3 (12.5%)	0.662
On antihypertensives	183 (88.4%)	165 (90.2%)	17 (73.9%)	0.022
On OAC/NOAC	53 (23.5%)	45 (22.4%)	8 (33.3%)	0.232
**Consciousness at arrival**				
Awake	193 (82.1%)	178 (84.8%)	14 (58.3%)	< 0.001
Reduced consciousness	19 (8.1%)	15 (7.1%)	4 (16.7%)	
Unconscious	12 (5.1%)	7 (3.3%)	6(25.0%)	
Unknown	11 (4.7%)	11 (5.2%)	0 (0.0%)	
CT performed	225 (99.1%)	200 (99.0%)	24 (100.0%)	0.624
MRI performed	15 (6.6%)	14 (6.9%)	1 (4.2%)	0.607
Thrombolysis	12 (5.3%)	12 (6.0%)	0 (0.0%)	
Thrombectomy	5 (2.4%)	5 (2.5%)	0 (0.0%)	0.631
**Discharged to**				
Nursing home	46 (19.6%)	39 (18.6%)	7 (29.2%)	0.001
Home	93 (39.6%)	90 (42.9%)	3 (12.5%)	
Rehab	35 (14.9%)	32 (15.2%)	2(12.3%)	
Other facility	25 (10.6%)	24 (11.4%)	0 (0%)	
Died	36 (15.3%)	25 (11.9%)	10 (41.7%)	
30-day mortality	46 (19.6%)	34 (16.2%)	11 (45.8%)	< 0.001
1-year mortality	93 (44.1%)	79 (41.8%)	13 (61.9%)	0.167
3-year mortality	123 (67.3%)	108 (66.2%)	14 (74.6%)	0.521
5-year mortality	133 (81.7%)	117 (80.5%)	n/a	

*IS* Ischemic stroke, *HS* haemorrhagic stroke, *TAVR* transcatheter aortic valve replacement, *TIA* transient ischemic attack, *ASA* acetylsalicylic acid, *(N) OAC* (NEW) oral anticoagulation, *CT* computed tomography, *MRI* magnetic resonance imaging.

After the stroke, almost half of the patients (39.6%) could be discharged home, and patients with IS were more often discharged home than patients with HS (42.9% versus 12.5%, p < 0.001).

**Figure 3:**
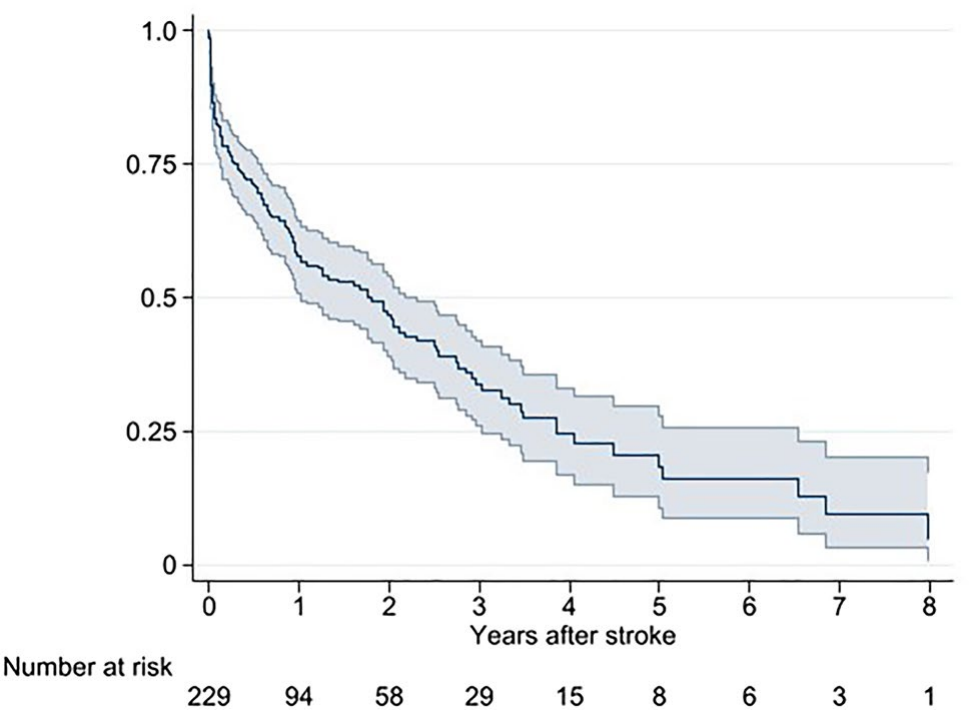
Survival after stroke presented as Kaplan–Meier survival function with 95% CI.

The 30-day mortality for stroke was 19.6%, where IS had a better 30-day survival than HS (16.2% vs 45.8%, p < 0.001). The 1-, 3-, and 5-year mortality was 44.1%, 67.3% and 81.7%, respectively, with no significant difference between HS and IS (Fig. 3, Supplementary Fig. 2, Table 2). Risk factors for 30-day mortality were level of consciousness at arrival, where unconsciousness had a OR of 19.8 (95% CI 8.26–47.5, p < 0.001) and reduced consciousness had a OR of 4.89 (2.17–11.0, p < 0.001, Supplementary Table 4). Risk factors for 1-year mortality were also level of consciousness at arrival, where unconscious had a OR of 30.8 (11.7–81.3, p < 0.001) and reduced consciousness had a OR of 4.79 (2.63–8.71, p < 0.001) together with thrombectomy performed 3.35 (1.20–9.33, p = 0.021, Supplementary Table 5).

Patients presenting with stroke were compared based on the presence of atrial fibrillation at arrival. Patients with atrial fibrillation were more often on anticoagulation (42.0% versus 5.2%, < 0.001), were less often on acetylsalicylic acid (25.0% versus 68.7%, < 0.001) and less often on clopidrogel (5.0% versus 25.2%, p < 0.001, Supplementary Table 6). There were no significant differences in the level of consciousness at arrival or survival at 30-day, 1, 3, and 5 years (Supplementary Table 6). At discharge, 38% of patients with atrial fibrillation were on warfarin, and 33% were on new oral anticoagulation (NOAC).

## Discussion

In this nationwide, registry-based all-comers study of late stroke after TAVR, we could demonstrate a stroke rate between 2–3% per year during long-term follow-up. The majority (89%) suffered an ischemic stroke, and the 30-day mortality was 20%.

One key question that we tried to address in this study was whether having a TAVR valve is associated with an increased risk of stroke. Patients receiving TAVR are usually old, have risk factors that could predispose them to stroke, and subsequently, already carry an increased background risk for stroke. Any attempts to address this question need to take this into account. By combining official population data to obtain the number of individuals for each age and sex, we used Riksstroke to calculate an estimated standardized incidence for each individual and compared it to the actual outcome in this cohort. The standardized incidence ratio varied between 1.15% and 1.75% during the follow-up. The intention was not to make a direct comparison but to get a perspective on the risk of stroke after TAVR. A direct comparison between the actual outcome and standardized incidence falls short for two reasons. Firstly, the diagnosis of stroke in the TAVR cohort was taken from two sources, where the National Patient Registry accounted for 3% of patients. For the standardized incidence estimation, only Riksstroke was used, since the NPR could not be accessed for the entire Swedish population. In the TAVR population, we found an underreporting of 3%, but validation studies have reported figures between 5 and 11% missing cases in the registry^21^. Secondly, the TAVR cohort had more comorbidities than the general population. In the present study, 37% of patients had atrial fibrillation, whereas the same age group in Sweden has a 21–24% prevalence of atrial fibrillation^22^. The group also had a 74% frequency of hypertension, whereas the general population has lower numbers (58–63%). Diabetes on the other hand was not overrepresented, with 24% prevalence compared to 21–25% prevalence in the general population^23^. Given that the TAVR cohort had a higher prevalence of comorbidities and that we have underestimated the standardized incidence slightly, the higher number of strokes in the TAVR cohort may be explained by these observations. However, we cannot exclude that the stent frame, the pericardial valve tissue, or immobilized native leaflets affects stroke rate. Given the observation that there was no difference between low frame valves (BEVs) and valves with frames expanding into the LVOT and ascending aorta (SEVs), this effect is probably not that large.

The present study identified reduced estimated GFR (eGFR < 30 ml/min/1.73 m^2^), diabetes, a history of stroke, patient age, and male sex as pre-disposing factors for stroke after TAVR. These findings are in concordance with other reports describing risk factors for stroke both in the general population and in the TAVR population^8,24^. Interestingly, a valve-in-valve procedure was associated with a reduced stroke rate. The only explanation for this unexpected finding could be that either valve-in-valve patients are generally healthier as the threshold for accepting them for TAVR is higher. In our cohort the valve-in-valve group were younger, had less peripheral vascular disease and were less often on steroids (Supplementary Table 8). Another explanation is that they received more intense anticoagulation after the procedure or that there are residual confounding factors. The model created yielded a Harrell's C-index of 0.64, which should be considered as relatively good given the heterogeneity of the material.

Almost 90% of the patients suffered an ischemic stroke, whereas 10% had a haemorrhagic stroke and in one patient, the stroke type was not known. Patients with ischemic stroke had more hypertension and presented with a higher level of consciousness at admission.

There were no statistical differences in anti-platelet therapy or anticoagulation between ischemic and haemorrhagic stroke. However, the tendency was that ischemic stroke patients were more often on acetylsalicylic acid and clopidrogel, whereas patients with haemorrhagic stroke were more often on anticoagulation. In the ischemic stroke group, 21% were not on any antithrombotic treatment, whereas in the haemorrhagic stroke group, 33% were not on any antithrombotic treatment. The databases do not provide any information on the indication for different antithrombotic treatment or the reason why patients had no treatment, which prevents us from a more detailed analysis. Given these figures, it is hard to get any indication as to whether the current regimen with acetylsalicylic acid in the long-term after TAVR is as appropriate as antithrombotic treatment.

The outcome after stroke showed an expected 30-day mortality of 20%. The short- and long-term survival was very much in line with what is seen after stroke in a corresponding age group in the general population in Sweden and in similar studies^25,26^. In terms of functional class, 40% could be discharged home, whereas 35% were discharged to a nursing home or rehab. There was some difference in outcome depending upon type of stroke. Patients with ischemic stroke were more often discharged home and had a better short-term prognosis. By far, the strongest predictor for outcome after stroke was level of consciousness at admission. This pattern also is seen in the general population^25^.

Atrial fibrillation is a strong pre-disposing and treatable causative factor for a stroke. This was seen in the univariable analysis but surprisingly not in the multivariable analysis. The plausible explanation for this is that we measure atrial fibrillation at the time of implant and have no records of atrial rhythm for all patients years later when the risk for stroke presents. It is reasonable to believe that quite a few patients have developed atrial fibrillation after the implant and not received adequate treatment. This hypothesis is supported by the fact that only 42% of patients with ischemic stroke were on OAC/NOAC. This observation warrants further studies on the low grade of anticoagulation in these stroke patients.

A retrospective registry-based study is limited by the amount and quality of data available. The strength of this study is its comprehensiveness, where all TAVR procedures in Sweden during the study period were included and where we were able to cross-reference the SWEDEHEART data with Riksstroke and National Patient Registry: three registries with a high level of completeness and accuracy^15,16,21,27^. The primary weakness is shared with all registry-based studies, i.e., incompleteness or inaccuracy of data depending on human factor or missing information. Despite a relatively large sample of 4000+ patients, a larger cohort would have yielded more robust data, but as all procedures in Sweden were included, a larger dataset was not possible. With the increasing number of TAVR, a study follow-up at 5 years would probably increase the number of individuals by a factor of three and would also have more individuals in the later follow-up period. We used the general population as bench-mark, but the best comparison would have been to create a cohort of SAVR patient that exactly matched our population. However, this is close to impossible to perform as most TAVR patients in this study were in prohibitive risk-category where no matching cases can be found in the SAVR population. Also, many patients are burdened by co-morbidities that do not show up in registries (frailty, malignancy, liver function cognitive function, etc.) and matching will inherently be skewed. One major limitation is the lack of data on type of indication for antithrombotic from the implantation of TAVR to follow-up. Despite combing three national quality registries of high quality, this data could not be obtained. Another issue that can be raised is the competing risk for death in this type of patient cohort. However, competing risk analysis did not change the spectrum of risk factors for stroke (Supplementary Table 7).

In this study, we could demonstrate an increased risk of stroke in this patient category, but it was unclear whether this was dependent on the TAVR valve per se or the higher frequency of pre-disposing risk factors in this cohort. In any case, the risk increase is modest and should not affect decision-making in the treatment of aortic valve stenosis.

## Supplementary Information

Supplementary Information.
