# Supplementary material for: Late stroke after transcatheter aortic valve replacement: a nationwide study
Source: Sci Rep. 2021 May 5;11:9593. doi: 10.1038/s41598-021-89217-0 (PMC8100158; doi:10.1038/s41598-021-89217-0)
Supplement: Supplementary file 1 — Supplementary Information. [file 41598_2021_89217_MOESM1_ESM.docx]

**Appendix**

**Late Stroke After Transcatheter Aortic Valve Replacement: A Nationwide Study**

SWEdish study on Stroke After TAVI (SWESTAT)

|  | **Name, Degree(s)** | **Institution, City, Country, email** |
| --- | --- | --- |
| 1 | Henrik Bjursten MD PhD | Lund University, Department of Cardiothoracic Surgery, Skåne University Hospital, Lund, Sweden.  [henrik.bjursten@med.lu.se](mailto:henrik.bjursten@med.lu.se) |
| 2 | Professor Bo Norrving MD PhD | Lund University, Department of Clinical Sciences Lund, Neurology, Skåne University Hospital, Lund, Sweden  bo.norrving@med.lu.se |
| 3 | Sigurdur Ragnarsson MD PhD | Lund University, Department of Cardiothoracic Surgery, Skåne University Hospital, Lund, Sweden.  [sigurdur.ragnarsson@med.lu.se](mailto:henrik.bjursten@med.lu.se) |

Content

[Data Sources 2](#_Toc23193753)

[SWENTRY registry 2](#_Toc23193754)

[NPR registry 2](#_Toc23193755)

[Riksstroke 2](#_Toc23193756)

[Method for estimating standardized incidence for the study cohort 4](#_Toc23193757)

[eTable 1 – Variables used in the analysis 6](#_Toc23193758)

[eTable 2 – Implantations per valve type and year 8](#_Toc23193759)

[eTable 3 – Hazard rate and standardized incidence for stroke after TAVI 9](#_Toc23193760)

[eTable 4 – Risk factors for 30-day death after stroke 10](#_Toc23193761)

[eTable 5 – Risk factors for 1-year death after stroke 11](#_Toc23193762)

[eTable 6 – Atrial fibrillation and stroke patients 12](#_Toc23193763)

[eTable 7 – Competing risk analysis 13](#_Toc23193764)

[eFigure 1 - Risk of developing stroke based on eGFR 14](#_Toc23193765)

[eFigure 2 – Survival after stroke based on ischemic or hemorrhagic stroke 15](#_Toc23193766)

[References 16](#_Toc23193767)

# Data Sources

## SWENTRY registry

The SWEDEHEART registry (Swedish Web system for Enhancement and Development of Evidence-based care in Heart disease Evaluated According to Recommended Therapies) was founded in 1992 and now contains information on all patients undergoing coronary angiography, percutaneous coronary intervention, transcatheter aortic valve replacement and cardiac surgery as well as patients admitted to the cardiac intensive care unit(1). In 2010, the SWENTRY registry (SWEdish traNscatheter cardiac intervention regisTRY) was added to the SWEDEHEART registry. As the first TAVI procedures were performed in 2008, data for the years 2008-2009 was entered retrospectively. From 2010, data have been added prospectively. The registry contains pre-procedural data, procedural data and outcome data at 30 days. All centres performing TAVI enter all procedures performed. This registry also is updated on a weekly basis against the national tax registry for deaths.

## NPR registry

The Swedish National Patient Register (NPR) is maintained by a government agency, the Swedish National Board of Health and Welfare, and was founded in 1987. Hospitals are mandated by law to enter all period of hospitalizations. Each entry includes a diagnosis following the International Classification of Diseases (ICD) codes, and up to 30 diagnoses can be entered. A diagnosis is recorded by a doctor during hospitalization and at the time of discharge. Interventions also are recorded according to the ICD classification.

The validity of many the diagnoses in this registry has been evaluated and was found to be 95% for a primary diagnosis of heart failure. The positive predictive value was 98.6% for stroke and 98% to 100% for myocardial infarction(2, 3).

## Riksstroke

The Riksstroke is a national registry covering all hospitalization for stroke. The registry was founded in 1994, and four years later, all hospitals managing stroke patients participated. Today, all 72 hospitals managing stroke patients actively enter data in the database. The completeness of data has been estimated between 89-95% (4). Data entry is continuously monitored and validated (5). The registry also is used as a tool for clinical follow-up after stroke. Every year an annual report is presented that covers stroke incidence, outcome, treatment regimens and quality at different regions of the country.

# Method for estimating standardized incidence for the study cohort

| **Step 1** |  |  |  |  |  |  |  |  |
| --- | --- | --- | --- | --- | --- | --- | --- | --- |
|  | **Number in population** | | | **Number suffering stroke** | | | **Standardized risk** | |
| Age | Men | Women |  | Men | Women |  | Men | Women |
| **20** | 56782 | 52151 |  | 1 |  |  | 0,00% | 0,00% |
| - | - | - |  | - | - |  | - | - |
| - | - | - |  | - | - |  | - | - |
| **76** | 37482 | 40689 |  | 356 | 277 |  | 0,95% | 0,68% |
| **77** | 34368 | 38033 |  | 417 | 303 |  | 1,21% | 0,80% |
| **78** | 32825 | 37459 |  | 410 | 328 |  | 1,25% | 0,88% |
| **79** | 29894 | 35393 |  | 356 | 285 |  | 1,19% | 0,81% |
| **80** | 27002 | 32489 |  | 356 | 322 |  | 1,32% | 0,99% |
| **81** | 24520 | 30728 |  | 359 | 332 |  | 1,46% | 1,08% |
| **82** | 22041 | 27919 |  | 319 | 355 |  | 1,45% | 1,27% |
| **Up to 99 year** | |  |  |  |  |  |  |  |
|  |  |  |  |  |  |  |  |  |
| **Step 2** |  |  |  |  |  |  |  |  |
| **Patient number** | **Sex** | **Age at implant** | **Year post TAVI** | |  |  |  |  |
|  |  |  | **1** | **2** | **3** | **4** | **5** | **6** |
| 1 | Male | 82 | 83 | 84 | 85 | 86 | * |  |
| 2 | Female | 77 | 78 | 79 | 80 | 81 | 82 | * |
| 3 | Male | 85 | 86 | 87 | * |  |  |  |
| - | - | - | - | - | - | - | - | - |
| 4205 | Female | 75 | 76 | 77 | * |  |  |  |
|  |  |  |  |  |  |  |  |  |
|  |  |  |  |  |  |  |  |  |
| **Step 3** |  |  |  |  |  |  |  |  |
| **Patient number** | **Sex** | **Year post TAVI** | |  |  |  |  |  |
|  |  | **0** | **1** | **2** | **3** | **4** | **5** | **6** |
| 1 | Male | 1,45% | 1,53% | 1,73% | 1,96% | 1,85% | * |  |
| 2 | Female | 0,80% | 0,88% | 0,81% | 0,99% | 1,08% | 1,27% | * |
| 3 | Male | 1,96% | 1,85% | 1,94% | * |  |  |  |
| - | - | - | - | - | - | - | - | - |
| 4205 | Female | 0,64% | 0,68% | 0,80% | * |  |  |  |
|  |  |  |  |  |  |  |  |  |
| **Standardized risk for the population** | | **1,46%** | **1,55%** | **1,65%** | **1,74%** | **1,78%** | **1,81%** | **1,93%** |
|  | |  |  |  |  |  |  |  |

**Step 1**: Census data on number of persons in each age group and sex is extracted from the national tax registry for 2017. Number of strokes for each age group and sex for 2017 is extracted from Riksstroke.

**Step 2:** The study cohort depicted with time in study for individuals and their age at each year post TAVI. |* denotes that the patient leaves the study (dead or censored).

**Step 3:** Each age/sex point is substituted with the corresponding risk from Step 1. A mean is calculated for the study population and used as standardized risk for comparison.

# eTable 1 – Variables used in the analysis

|  | n | All | No Stroke  n=3970 | Stroke  n=235 | P-value | 95% CI |
| --- | --- | --- | --- | --- | --- | --- |
| Age (years) | 4205 | 81.5 ± 7.4 | 81.4 ± 7.4 | 82.3 ± 7.1 | 0.066 | 1.02 (1.00-1.04)* |
| Male sex | 4202 | 2150 (51.2%) | 2022 (51.0%) | 128 (54.5%) | 0.096 | 1.24 (0.96-1.61)* |
| Height (cm) | 4191 | 168.0 ± 9.5 | 168.0 ± 9.5 | 167.6 ± 9.4 | 0.784 | 1.00 (0.98-1.01) |
| Weight (kg) | 4200 | 75.1 ± 16.7 | 75.2 ± 16.8 | 73.5 ± 15.6 | 0.162 | 0.99 (0.99-1.00) |
| BSA (m2)† | 4191 | 1.8 ± .2 | 1.8 ± 0.2 | 1.8 ± 0.2 | 0.252 | 0.70 (0.38-1.29) |
| Body mass index | 4191 | 27.6 ± 3.1 | 27.6 ± 3.1 | 27.7 ± 3.1 | 0.804 | 1.01 (0.97-1.05) |
| Serum creatinine (µmol/L) | 4204 | 106.6 ± 65.6 | 106.0 ± 64.0 | 116.9 ± 88.4 | 0.002 | 1.00 (1.00-1.00) |
| eGFR (ml/min/1.73 m2) † | 4201 | 57.5 ± 19.3 | 57.6 ± 19.2 | 56.0 ± 21.1 | 0.069 | 0.99 (0.99-1.00) |
| eGFR<30 ml/min/1.73 m2 † | 4201 | 313 (7.5%) | 286 (7.2%) | 27 (11.5%) | 0.001 | 2.03 (1.36-3.03)* |
| eGFR<60 ml/min/1.73 m2 † | 4201 | 2289 (54.5%) | 2159 (54.4%) | 130 (55.3%) | 0.540 | 1.08 (0.84-1.40) |
| Hypertension | 4204 | 3113 (74.0%) | 2931 (73.8%) | 182 (77.4%) | 0.173 | 1.24 (0.91-1.68) |
| Diabetes | 4204 | 1019 (24.2%) | 946 (23.8%) | 73 (31.1%) | 0.001 | 1.59 (1.20-2.09)* |
| Previous cardiac surgery | 4205 | 989 (23.5%) | 928 (23.4%) | 61 (26.0%) | 0.454 | 0.89 (0.67-1.20) |
| Myocardial infarction 3 months prior to procedure | 4205 | 235 (5.6%) | 219 (5.5%) | 16 (6.8%) | 0.310 | 1.30 (0.78-2.16) |
| Previous PCI | 4204 | 1148 (27.3%) | 1076 (27.1%) | 72 (30.6%) | 0.503 | 1.10 (0.83-1.45) |
| COPD | 4205 | 827 (19.7%) | 784 (19.7%) | 43 (18.3%) | 0.826 | 0.96 (0.69-1.34) |
| Previous stroke | 4205 | 568 (13.5%) | 522 (13.1%) | 46 (19.6%) | 0.004 | 1.60 (1.16-2.21)* |
| Critical preoperative state | 4205 | 125 (3.0%) | 116 (2.9%) | 9 (3.8%) | 0.173 | 1.59 (0.82-3.09) |
| Peripheral vascular disease | 4204 | 798 (19.0%) | 743 (18.7%) | 55 (23.4%) | 0.056 | 1.34 (0.99-1.82)* |
| Reduced mobility | 4205 | 593 (14.1%) | 559 (14.1%) | 34 (14.5%) | 0.229 | 1.25 (0.87-1.80) |
| Atrial fibrillation | 4205 | 1561 (37.1%) | 1482 (37.3%) | 79 (33.6%) | 0.803 | 0.97 (0.74-1.27)* |
| Porcelain aorta | 4204 | 85 (2.0%) | 73 (1.8%) | 12 (5.1%) | 0.299 | 1.36 (0.76-2.45)* |
| Steroid treatment | 4202 | 341 (8.1%) | 327 (8.2%) | 14 (6.0%) | 0.285 | 0.74 (0.43-1.28) |
| History of malignancy | 4199 | 447 (10.6%) | 420 (10.6%) | 27 (11.5%) | 0.492 | 1.15 (0.77-1.72) |
| History of radiation treatment | 4204 | 34 (0.8%) | 30 (0.8%) | 4 (1.7%) | 0.643 | 1.26 (0.47-3.40) |
| Mean aortic gradient (mmHg) | 4185 | 47.6 ± 15.6 | 47.6 ± 15.6 | 48.6 ± 15.5 | 0.711 | 1.00 (0.99-1.01) |
| AVA (cm2) | 4090 | 0.66 ± 0.21 | 0.66 ± 0.21 | 0.64 ± 0.19 | 0.317 | 0.72 (0.38-1.36) |
| LVEF normal | 4205 | 2582 (61.4%) | 2443 (61.5%) | 139 (59.1%) |  | Reference |
| LVEF slightly depressed | 4205 | 726 (17.3%) | 677 (17.1%) | 49 (20.9%) | 0.079 | 1.34 (0.97-1.86)* |
| LVEF moderately depressed | 4205 | 558 (13.3%) | 528 (13.3%) | 30 (12.8%) | 0.780 | 1.06 (0.71-1.57) |
| LVEF severely depressed | 4205 | 333 (7.9%) | 316 (8.0%) | 17 (7.2%) | 0.963 | 1.01 (0.61-1.68) |
| **Procedural variables** |  |  |  |  |  |  |
| Contrast (ml) | 4192 | 84.8 ± 65.2 | 84.1 ± 65.1 | 96.6 ± 66.7 | 0.873 | 1.00 (1.00-1.00) |
| Flouro time (sec) | 4171 | 1269± 845 | 1264± 847 | 1342± 804 | 0.780 | 1.00 (1.00-1.00) |
| Transfemoral access | 4205 | 3621 (86.1%) | 3433 (86.5%) | 188 (80.0%) |  | Reference |
| Transapical access | 4205 | 405 (9.6%) | 370 (9.3%) | 35 (14.9%) | 0.166 | 1.29 (0.90-1.86) |
| Direct aortic access | 4205 | 80 (1.9%) | 76 (1.9%) | 4 (1.7%) | 0.922 | 1.05 (0.39-2.83) |
| Subclavian access | 4205 | 99 (2.4%) | 91 (2.3%) | 8 (3.4%) | 0.187 | 1.61 (0.79-3.27) |
| Device SEV † | 4205 | 2209 (52.5%) | 2088 (52.6%) | 121 (51.5%) | 0.955 | 1.01 (0.78-1.30)* |
| Valve-in-valve | 4205 | 177 (4.0%) | 176 (4.4%) | 1 (0.4%) | 0.017 | 0.09 (0.01-0.65) |
| Predilatation | 3605 | 1700 (47.2%) | 1631 (47.5%) | 69 (39.7%) | 0.416 | 0.88 (0.65-1.20) |
| Postdilatation | 4202 | 734 (17.5%) | 699 (17.6%) | 35 (14.9%) | 0.989 | 1.00 (0.70-1.44) |
| Second valve implanted | 4204 | 125 (3.0%) | 116 (2.9%) | 9 (3.8%) | 0.896 | 1.05 (0.54-2.04) |
| Intubation anesthesia | 4205 | 1662 (39.5%) | 1557 (39.2%) | 105 (44.7%) | 0.801 | 0.97 (0.75-1.25) |
| Heart-lung machine (unplanned) | 4205 | 24 (0.6%) | 22 (0.6%) | 2 (0.9%) | 0.663 | 0.73 (0.18-2.95) |
| Acute vascular surgery | 4205 | 7 (0.2%) | 6 (0.2%) | 1 (0.4%) | 0.304 | 2.80 (0.39-20.0) |
| Acute conversion to cardiac surgery | 4205 | 60 (1.4%) | 59 (1.5%) | 1 (0.4%) | 0.257 | 0.32 (0.05-2.29) |
| **Outcome 30 Days** |  |  |  |  |  |  |
| New atrial fibrillation | 4205 | 58 (1.4%) | 50 (1.3%) | 8 (3.4%) | 0.111 | 1.78 (0.88-3.60)* |
| Vascular complication | 4204 | 89 (2.1%) | 82 (2.1%) | 7 (3.0%) | 0.249 | 1.56 (0.73-3.31) |
| Myocardial infarction | 4204 | 27 (0.6%) | 26 (0.7%) | 1 (0.4%) | 0.946 | 0.93 (0.13-6.66) |
| New permanent pacemaker | 4205 | 252 (6.0%) | 199 (5.0%) | 19 (8.0%) | 0.964 | 1.01 (0.63-1.62) |
| Bleeding needing treatment | 4205 | 101 (2.4%) | 97 (2.4%) | 4 (1.7%) | 0.205 | 0.53 (0.20-1.42) |
| Infection needing treatment | 4204 | 170 (4.0%) | 157 (4.0%) | 13 (5.6%) | 0.406 | 1.27 (0.72-2.22) |
| New dialysis | 4204 | 20 (0.5%) | 18 (0.5%) | 2 (0.9%) | 0.098 | 3.24 (0.81-13.1)* |
| Aortic regurgitation - grade I-III† | 4205 | 1926 (45.8%) | 1812 (45.6%) | 114 (48.5%) | 0.362 | 0.89 (0.69-1.15) |
| Aortic regurgitation - grade II-III† | 4205 | 216 (5.1%) | 203 (5.1%) | 13 (5.5%) | 0.827 | 0.94 (0.52-1.68) |

**eTable 1**: Variables from the SWENTRY registry used in the analysis presented both for the whole as well as grouped on the presence of PVE and described as mean ±SD, number (%) or median (IQR). P-levels and HR from Univariable Cox-regression. eGFR= Estimated glomerular filtration rate. PCI=percutaneous coronary intervention. COPD=chronic obstructive pulmonary disease. AVA=aortic valve area. LVEF=left ventricular ejection fraction. SEV=Self expanding valve. Variables denoted with * were entered in the multivariable analysis. † variables calculated form other variables in the SWENTRY registry.

# eTable 2 – Implantations per valve type and year

| **Valve** | **Year** |  |  |  |  |  |  |  |  |  |  |  |
| --- | --- | --- | --- | --- | --- | --- | --- | --- | --- | --- | --- | --- |
| **Mechanical/Balloon Expandable (MEV/BEV)** | 2008 | 2009 | 2010 | 2011 | 2012 | 2013 | 2014 | 2015 | 2016 | 2017 | 2018 | **Total** |
| Boston Lotus |  |  |  |  |  | 9 | 38 | 78 | 79 | 7 |  | **211** |
| Edwards Sapien 3 |  |  |  |  |  |  |  | 22 | 294 | 374 | 232 | **922** |
| Edwards Sapien 3 Ultra |  |  |  |  |  |  |  |  |  |  | 2 | **2** |
| Edwards Sapien / Sapien XT | 25 | 64 | 70 | 93 | 104 | 137 | 166 | 194 | 7 |  | 1 | **861** |
| **Self-expanding (SEV)** |  |  |  |  |  |  |  |  |  |  |  |  |
| Abbot Portico |  |  |  |  |  | 1 | 11 |  | 32 | 13 | 32 | **89** |
| JenaValve |  |  |  |  |  |  | 1 |  |  |  |  | **1** |
| Medtronic CoreValve / Evolut | 49 | 68 | 67 | 119 | 159 | 161 | 196 | 162 | 16 | 1 | 2 | **1000** |
| Medtronic Evolut PRO |  |  |  |  |  |  |  |  |  | 10 | 25 | **35** |
| Medtronic Evolut R |  |  |  |  |  |  |  | 77 | 135 | 275 | 175 | **662** |
| Symetis Acurate neo/TF |  |  |  |  |  |  |  | 22 | 79 | 145 | 176 | **421** |
| Symetis Acurate TA |  |  |  |  |  |  |  |  |  |  | 3 | **3** |
| **Total** | **74** | **132** | **137** | **212** | **263** | **308** | **412** | **555** | **642** | **825** | **648** | **4208** |

**eTable 2:** Boston Lotus and Symetis Acurate are trademarks of Boston Scientific (Boston. MA). Edwards Sapien, Sapien XT, Sapien 3 and Sapien Ultra are trademarks of Edwards Lifesciences (Irvine. Ca). Abbot Portico is a trademark of Abbot Vascular (Santa Clara, CA). Jenavalve is a trademark of JenaValve Technology (Munich, Germany). Medtronic Corevalve and Evolute are trademarks of Medtronic Inc (Minneapolis, MN).

# eTable 3 – Hazard rate and standardized incidence for stroke after TAVI

| **Year** | **Hazard Rate** | **Lo CI** | **Hi CI** |  | **Standardized incidence** | **Standardized Incidence ratio** |
| --- | --- | --- | --- | --- | --- | --- |
| 0-1 | 2.00% | 1.54% | 2.46% |  | 1.46% | 1.37 |
| 1-2 | 2.23% | 1.65% | 2.80% |  | 1.55% | 1.43 |
| 2-3 | 2.12% | 1.44% | 2.80% |  | 1.65% | 1.29 |
| 3-4 | 2.76% | 1.77% | 3.75% |  | 1.74% | 1.59 |
| 4-5 | 3.12% | 1.75% | 4.48% |  | 1.78% | 1.75 |
| 5-6 | 2.09% | 0.64% | 3.53% |  | 1.81% | 1.15 |
| 6-7 | 2.31% | 0.29% | 4.33% |  | 1.93% | 1.20 |
| 7-8 | 2.75% | -0.36% | 5.87% |  | 1.88% | 1.46 |
| 8-9 | 2.08% | -2.00% | 6.17% |  | 1.85% | 1.13 |

eTable 3: Hazard rate for stroke for the cohort together with a standardized incidence (and ratio) estimated for this cohort.

# eTable 4 – Risk factors for 30-day death after stroke

|  |  |  |  | Univariable |  | | | Multivariable | |
| --- | --- | --- | --- | --- | --- | --- | --- | --- | --- |
|  | **Alive (n=184)** | **Dead (n=44)** | **p-value** | | | **OR (95% CI)** | **p-value** | | **OR (95% CI)** |
| Age | 83.8 (7.6) | 83.9 (7.0) |  | 0.908 | 1.00 (0.96-1.05) | | |  |  |
| Male sex | 103 (56.0%) | 22 (50.0%) |  | 0.475 | 0.79 (0.41-1.52) | | |  |  |
| TIA/Amaurosis Fugax | 23 (12.6%) | 4 (9.3%) |  | 0.554 | 0.71 (0.23-2.18) | | |  |  |
| Previous stroke | 44 (24.0%) | 10 (22.7%) |  | 0.854 | 0.93 (0.42-2.03) | | |  |  |
| Atrial fibrillation | 91 (48.1%) | 21 (45.7%) |  | 0.761 | 0.90 (0.47-1.73) | | |  |  |
| Hypertension | 143 (78.6%) | 31 (72.1%) |  | 0.363 | 0.70 (0.33-1.50) | | |  |  |
| Diabetes | 62 (33.9%) | 19 (44.2%) |  | 0.207 | 1.55 (0.79-3.04) | | |  |  |
| On lipids | 92 (50.5%) | 22 (50.0%) |  | 0.948 | 0.98 (0.51-1.89) | | |  |  |
| On ASA | 87 (47.8%) | 20 (45.5%) |  | 0.780 | 0.91 (0.47-1.76) | | |  |  |
| On clopidrogel | 25 (13.7%) | 10 (22.7%) |  | 0.143 | 1.85 (0.81-4.20) | | |  |  |
| On antihypertensives | 148 (89.2%) | 35 (85.4%) |  | 0.499 | 0.71 (0.26-1.92) | | |  |  |
| On OAC/NOAC | 43 (23.6%) | 10 (22.7%) |  | 0.899 | 0.95 (0.43-2.08) | | |  |  |
| Thrombolysis | 10 (5.5%) | 2 (4.5%) |  | 0.801 | 0.82 (0.17-3.88) | | |  |  |
| Thrombectomy | 5 (2.9%) | 0 (0.0%) |  | 0.999 | 0.00 (0.00-0.00) | | |  |  |
| Level of consciousness |  |  |  |  |  | | |  |  |
| Unknown | 8 (4.2%) | 3 (6.5%) |  | 0.145 | 2.44 (0.72-8.16) | | | 0.145 | 2.44 (0.72-8.16) |
| Unconscious | 0 (0.0%) | 12 (26.1%) | <0.001 | | | 19.8 (8.26-47.5) | <0.001 | | 19.8 (8.26-47.5) |
| Decreased | 11 (5.8%) | 8 (17.4%) |  | <0.001 | 4.89 (2.17-11.0) | | | <0.001 | 4.89 (2.17-11.0) |
| Awake | 170 (89.9%) | 23 (50.0%) |  | Reference |  | | | Reference |  |

**eTable 4**: Risk factors for 30-day death after stroke. TIA=Transient Ischemic Attack. ASA= acetylsalicylic acid. (N)OAC=(New) Oral Anticoagulation.

# eTable 5 – Risk factors for 1-year death after stroke

|  |  |  |  | Univariable | | | Multivariable | |  |
| --- | --- | --- | --- | --- | --- | --- | --- | --- | --- |
|  | **Alive (n=138)** | **Dead (n=90)** | | **p-value** | **OR (95% CI)** | **p-value** | **OR (95% CI)** |  |  |
| Age | 83.7 (7.0) | 83.9 (8.2) |  | 0.848 | 1.00 (0.97-1.04) |  |  |  |  |
| Male sex | 78 (56.5%) | 47 (52.2%) |  | 0.524 | 0.84 (0.49-1.43) |  |  |  |  |
| TIA/Amaurosis Fugax | 14 (10.2%) | 13 (14.6%) |  | 0.323 | 1.50 (0.67-3.37) |  |  |  |  |
| Previous stroke | 34 (24.8%) | 20 (22.2%) |  | 0.653 | 0.87 (0.46-1.63) |  |  |  |  |
| Atrial fibrillation | 61 (43.0%) | 51 (54.8%) |  | 0.075 | 1.61 (0.95-2.73) |  |  |  |  |
| Hypertension | 110 (80.9%) | 64 (71.9%) |  | 0.118 | 0.61 (0.32-1.14) |  |  |  |  |
| Diabetes | 46 (33.6%) | 35 (39.3%) |  | 0.379 | 1.28 (0.74-2.23) |  |  |  |  |
| On lipids | 76 (55.9%) | 38 (42.2%) |  | 0.045 | 0.58 (0.34-0.99) |  |  |  |  |
| On ASA | 67 (49.3%) | 40 (44.4%) |  | 0.478 | 0.82 (0.48-1.41) |  |  |  |  |
| On clopidrogel | 21 (15.4%) | 14 (15.6%) |  | 0.981 | 1.01 (0.48-2.11) |  |  |  |  |
| On antihypertensives | 108 (88.5%) | 75 (88.2%) |  | 0.949 | 0.97 (0.41-2.31) |  |  |  |  |
| On OAC/NOAC | 25 (18.4%) | 28 (31.1%) |  | 0.028 | 2.01 (1.08-3.74) |  |  |  |  |
| Thrombolysis | 9 (6.6%) | 3 (3.3%) |  | 0.290 | 0.49 (0.13-1.85) |  |  |  |  |
| Thrombectomy | 1 (0.8%) | 4 (4.9%) |  | 0.096 | 6.51 (0.71-59.33) | 0.021 | 3.35 (1.20-9.33) |  |  |
| Level of consciousness |  |  |  |  |  |  |  |  |  |
| Unknown | 7 (4.9%) | 4 (4.3%) |  | 0.744 | 1.18 (0.43-3.26) | 0.867 | 1.18 (0.16-8.57) |  |  |
| Unconscious | 0 (0.0%) | 12 (12.9%) | | <0.001 | 19.0 (8.08-44.6) | <0.001 | 30.8 (11.7-81.3) |  |  |
| Decreased | 3 (2.1%) | 16 (17.2%) | | <0.001 | 5.00 (2.86-8.74) | <0.001 | 4.79 (2.63-8.71) |  |  |
| Awake | 132 (93.0%) | 61 (65.6%) | | Reference | | | Reference | | |

**eTable 5**: Risk factors for 1-year death after stroke. TIA=Transient Ischemic Attack. ASA= acetylsalicylic acid. (N)OAC=(New) Oral Anticoagulation.

# eTable 6 – Atrial fibrillation and stroke patients

|  | **All (n=235)** | **With Afib (n=112)** | **No Afib (n=123)** | |
| --- | --- | --- | --- | --- |
| Age | 83.8 (7.5) | 84.1 (6.4) | 83.5 (8.4) | 0.552 |
| Male sex | 125 (54.8%) | 60 (54.0%) | 65 (56.0%) | 0.709 |
| I63 | 210 (89.4%) | 97 (87.0%) | 112 (91.1%) | 0.377 |
| I61 | 24 (10.2%) | 13 (12.0%) | 11 (8.9%) | 0.501 |
| TIA/Amaurosis Fugax | 27 (11.9%) | 6 (5.0%) | 21 (18.4%) | 0.002 |
| Previous stroke | 54 (23.8%) | 28 (25.0%) | 26 (22.6%) | 0.672 |
| Atrial fibrillation | 112 (47.7%) | 112 (100.0%) | 0 (0.0%) |  |
| Hypertension | 174 (77.3%) | 79 (71.0%) | 95 (83.3%) | 0.029 |
| Diabetes | 81 (35.8%) | 38 (34.0%) | 43 (37.7%) | 0.552 |
| On lipids | 114 (50.4%) | 58 (52.0%) | 56 (48.7%) | 0.593 |
| On ASA | 107 (47.3%) | 28 (25.0%) | 79 (68.7%) | <0.001 |
| On clopidrogel | 35 (15.5%) | 6 (5.0%) | 29 (25.2%) | <0.001 |
| On antihypertensives | 183 (88.4%) | 94 (90.0%) | 89 (86.4%) | 0.372 |
| On OAC/NOAC | 53 (23.5%) | 47 (42.0%) | 6 (5.2%) | <0.001 |
| Thrombolysis | 12 (5.3%) | 3 (3.0%) | 9 (7.9%) | 0.080 |
| Thrombectomy | 5 (2.4%) | 5 (5.0%) | 0 (0.0%) | 0.020 |
| 30-day mortality | 46 (19.6%) | 21 (19.0%) | 25 (20.3%) | 0.761 |
| 1-year mortality | 93 (39.6%) | 52 (46.0%) | 42 (34.1%) | 0.075 |
| 3-year mortality | 123 (52.3%) | 59 (53.0%) | 64 (52.0%) | 0.921 |
| 5-year mortality | 133 (56.6%) | 64 (57.0%) | 69 (56.1%) | 0.872 |
| On OAC at discharge | 44 (23.4%) | 35 (38.0%) | 9 (9.4%) | <0.001 |
| On NOAC at discharge | 33 (17.1%) | 30 (33.3%) | 3 (2.8%) | <0.001 |
| Consciousness |  |  |  |  |
| Unknown | 11 (4.7%) | 8 (6.5%) | 3 (2.7%) | 0.198 |
| Unconscious | 12 (5.1%) | 8 (6.5%) | 4 (3.6%) |  |
| Decreased | 19 (8.1%) | 7 (5.7%) | 12 (10.7%) |  |
| Awake | 193 (82.1%) | 100 (81.3%) | 93 (83.0%) |  |

**eTable 6**: A comparison between stroke patients presenting with or without atrial fibrillation. TIA=Transient Ischemic Attack. ASA= acetylsalicylic acid. (N)OAC=(New) Oral Anticoagulation.

# eTable 7 – Competing risk analysis

|  | **Multivariable** |  | **Competing Risk** | |  |
| --- | --- | --- | --- | --- | --- |
|  | **p-value** | **HR (95% CI)** | **p-value** | **HR (95% CI) Subhazard** | |
| eGFR<30 ml/min/1.73 m2 † | 0.000 | 2.07 (1.39-3.11) | 0.026 | 1.58 (1.06-2.37) | |
| Diabetes | 0.002 | 1.59(1.19-2.12) | 0.006 | 1.46 (1.11-1.93) | |
| History of stroke | 0.018 | 1.48 (1.07-2.05) | 0.012 | 1.51 (1.09-2.10) | |
| Age (per year) | 0.010 | 1.03 (1.00-1.05) | 0.043 | 1.02 (1.00-1.04) | |
| Male sex | 0.058 | 1.28 (0.99-1.67) | 0.152 | 1.21 (0.93-1.56) | |
| Valve-in-valve | 0.015 | 0.09 (0.01-0.62) | 0.017 | 0.09 (0.01-0.65) | |

eTable 7: The multivariable model for stroke together with a competing risk analysis for death.

# eTable 8 – Comparison demographics native valve and valve-in-Valve

|  | **Native valve**  **(n=4028)** | **VinV**  **(n=177)** |  |
| --- | --- | --- | --- |
| Age (years) | 81.7 (7.1) | 76.4 (10.3) | <0.001 |
| Male gender | 2049 (50.9%) | 101 (57.1%) | 0.921 |
| Height (cm) | 167.9 (9.5) | 169.0 (9.0) | 0.384 |
| Weight (kg) | 75.0 (16.7) | 78.0 (17.3) | 0.496 |
| BSA (m2)† | 1.8 (0.2) | 1.9 (0.2) | 0.787 |
| Body mass index | 27.6 (3.2) | 27.2 (2.9) | 0.206 |
| Serum creatinine (µmol/L) | 105.7 (61.9) | 127.7 (121.1) | <0.001 |
| eGFR (ml/min/1.73 m2) † | 57.6 (19.2) | 55.7 (21.7) | 0.016 |
| eGFR<30 ml/min/1.73 m2 † | 297 (7.4%) | 16 (9.0%) | 0.067 |
| eGFR<60 ml/min/1.73 m2 † | 2185 (54.3%) | 104 (58.8%) | 0.893 |
| Hypertension | 2992 (74.3%) | 121 (68.4%) | 0.211 |
| Diabetes | 983 (24.4%) | 36 (20.3%) | 0.276 |
| Previous cardiac surgery | 825 (20.5%) | 177 (100%) | <0.001 |
| Myocardial infarction 3 months prior to procedure | 228 (5.7%) | 7 (4.0%) | 0.004 |
| Previous PCI | 1113 (27.6%) | 35 (19.9%) | 0.054 |
| COPD | 802 (19.9%) | 25 (14.1%) | 0.020 |
| Previous stroke | 548 (13.6%) | 20 (11.3%) | 0.178 |
| Critical preoperative state | 108 (2.7%) | 17 (9.6%) | <0.001 |
| Peripheral vascular disease | 778 (19.3%) | 20 (11.3%) | <0.001 |
| Reduced mobility | 574 (14.3%) | 19 (10.7%) | 0.038 |
| Atrial fibrillation | 1488 (36.9%) | 73 (41.2%) | 0.654 |
| Porcelain aorta | 85 (2.1%) | 0 (0.0%) | 1.000 |
| Steroid treatment | 333 (8.3%) | 8 (4.5%) | <0.001 |
| History of malignancy | 423 (10.5%) | 24 (13.6%) | 0.030 |
| History of radiation treatment | 34 (0.8%) | 0 (0.0%) | 1.000 |
| Mean aortic gradient (mmHg) | 47.9 (15.3) | 42.0 (19.4) | <0.001 |
| AVA (cm2) | 0.66 (0.2) | 0.64 (0.4) | <0.001 |
| LVEF normal | 2581 (65.9%) | 111 (63.9%) | 0.555 |
| LVEF slightly depressed | 2489 (61.8%) | 93 (52.5%) | 0.266 |
| LVEF moderately depressed | 691 (17.2%) | 35 (19.8%) | 0.001 |
| LVEF severely depressed | 523 (13.%) | 35 (19.8%) | 0.942 |

**eTable** : Demographics for patient with a native or biological (Valve.in-Valve) aortic valve described as mean ±SD, number (%) or median (IQR). eGFR= Estimated glomerular filtration rate. PCI=percutaneous coronary intervention. COPD=chronic obstructive pulmonary disease. AVA=aortic valve area. LVEF=left ventricular ejection fraction.

# eFigure 1 - Risk of developing stroke based on eGFR

**eFigure 1**: Kaplan Meier Failure estimates where the cohort have been divided if eGFR<30 (red line= or more (blue line).

# eFigure 2 – Survival after stroke based on ischemic or hemorrhagic stroke

**eFigure 2**: Kaplan Meier survival curve, where the cohort has been dived into ischemic stroke (blueline) and hemorrhagic stroke (red line).

# References

1. Jernberg T, Attebring MF, Hambraeus K, Ivert T, James S, Jeppsson A, et al. The Swedish Web-system for enhancement and development of evidence-based care in heart disease evaluated according to recommended therapies (SWEDEHEART). Heart. 2010;96(20):1617-21.

2. Ludvigsson JF, Andersson E, Ekbom A, Feychting M, Kim JL, Reuterwall C, et al. External review and validation of the Swedish national inpatient register. BMC Public Health. 2011;11:450.

3. Ingelsson E, Arnlov J, Sundstrom J, Lind L. The validity of a diagnosis of heart failure in a hospital discharge register. Eur J Heart Fail. 2005;7(5):787-91.

4. Koster M, Asplund K, Johansson A, Stegmayr B. Refinement of Swedish administrative registers to monitor stroke events on the national level. Neuroepidemiology. 2013;40(4):240-6.

5. Soderholm A, Stegmayr B, Glader EL, Asplund K, Riksstroke C. Validation of Hospital Performance Measures of Acute Stroke Care Quality. Riksstroke, the Swedish Stroke Register. Neuroepidemiology. 2016;46(4):229-34.
